# Low Density Lipoprotein Receptor-Related Protein 1 Dependent Endosomal Trapping and Recycling of Apolipoprotein E

**DOI:** 10.1371/journal.pone.0029385

**Published:** 2012-01-04

**Authors:** Alexander Laatsch, Malamatenia Panteli, Marijke Sornsakrin, Britta Hoffzimmer, Thomas Grewal, Joerg Heeren

**Affiliations:** 1 Department of Biochemistry and Molecular Cell Biology, University Medical Center Hamburg-Eppendorf, Hamburg, Germany; 2 Faculty of Pharmacy A15, The University of Sydney, Sydney, Australia; Institut Curie, France

## Abstract

**Background:**

Lipoprotein receptors from the low density lipoprotein (LDL) receptor family are multifunctional membrane proteins which can efficiently mediate endocytosis and thereby facilitate lipoprotein clearance from the plasma. The biggest member of this family, the LDL receptor-related protein 1 (LRP1), facilitates the hepatic uptake of triglyceride-rich lipoproteins (TRL) via interaction with apolipoprotein E (apoE). In contrast to the classical LDL degradation pathway, TRL disintegrate in peripheral endosomes, and core lipids and apoB are targeted along the endocytic pathway for lysosomal degradation. Notably, TRL-derived apoE remains within recycling endosomes and is then mobilized by high density lipoproteins (HDL) for re-secretion. The aim of this study is to investigate the involvement of LRP1 in the regulation of apoE recycling.

**Principal Findings:**

Immunofluorescence studies indicate the LRP1-dependent trapping of apoE in EEA1-positive endosomes in human hepatoma cells. This processing is distinct from other LRP1 ligands such as RAP which is efficiently targeted to lysosomal compartments. Upon stimulation of HDL-induced recycling, apoE is released from LRP1-positive endosomes but is targeted to another, distinct population of early endosomes that contain HDL, but not LRP1. For subsequent analysis of the recycling capacity, we expressed the full-length human LRP1 and used an RNA interference approach to manipulate the expression levels of LRP1. In support of LRP1 determining the intracellular fate of apoE, overexpression of LRP1 significantly stimulated HDL-induced apoE recycling. Vice versa LRP1 knockdown in HEK293 cells and primary hepatocytes strongly reduced the efficiency of HDL to stimulate apoE secretion.

**Conclusion:**

We conclude that LRP1 enables apoE to accumulate in an early endosomal recycling compartment that serves as a pool for the intracellular formation and subsequent re-secretion of apoE-enriched HDL particles.

## Introduction

TRL, namely intestinal chylomicrons (CM) and liver-derived very low density lipoproteins (VLDL), deliver dietary and endogenous lipids through the bloodstream where fatty acids are liberated from triglycerides (TG) by the action of lipoprotein lipase (LPL). It is well established that released fatty acids are taken up by peripheral organs such as muscle, heart and white adipose tissue for energy storage or combustion [1]. Recently we showed that also activated brown adipose tissue drastically accelerates the clearance of triglycerides, a process crucially dependent on local LPL activity [2]. During lipolysis TRL remnant particles become enriched with HDL-derived apoE, and LPL remains associated with these particles (for review see [3]). These TRL remnants are rapidly cleared by the liver in an insulin-dependent manner via binding of apoE and LPL to LRP1 or heparan sulfate proteoglycans (HSPG) [4]–[9]. VLDL remnants are cleared via apoB100 and apoE binding to the LDL receptor (LDLR) (for review see [3], [10]). These processes involve an initial binding of TRL to HSPG or the scavenger receptor class B, type I (SRBI) before subsequent LDLR- and LRP1-mediated internalization [11], [12].

After receptor-mediated endocytosis the intracellular processing of TRL is quite complex and distinct from the classical LDL pathway. It could be demonstrated earlier that TRL disintegrate in peripheral endosomes, followed by a differential sorting of TRL components [13]–[16]. The majority of TRL lipids are targeted to lysosomes, whereas TRL-derived apoE and cholesterol accumulate in peripheral recycling endosomes [17]. Substantial amounts of TRL-derived apoE are then recycled back to the cell surface, re-secreted and found associated with HDL [13], [15]. We and others showed that HDL stimulated apoE recycling serves as an acceptor for TRL-derived apoE [15], [17], [18]. This process is associated with cholesterol efflux in hepatocytes and fibroblasts, and involves the internalization of HDL to endosomes containing TRL-derived apoE [17]. Most intriguingly, HDL-induced recycling of TRL-derived apoE4 is impaired compared to apoE3. Furthermore, reduced apoE4 recycling is associated with a decrease in cholesterol efflux in hepatocytes and fibroblasts [19]. However apoE4 recycling seems not to be linked to cholesterol efflux in neuronal cell lines [20]. Given the different metabolic properties of apoE isoforms, these findings could possibly be related to the development of atherosclerosis and Alzheimer's disease [3], [21], [22].

The role of lipoprotein receptors in TRL uptake and endosomal trafficking of TRL-derived apoE has been investigated in several studies [6], [7], [11], [12], [14], [16], [23]. However, little is known about the involvement of LRP1 in the regulation of apoE recycling. Since the secretion of TRL-derived apoE is not impaired in FH (familial hypercholesterolemia) fibroblasts lacking LDLR, we suggested that LRP1 might be responsible for the recycling process [13]. Moreover, LRP1 is essential for endocytosis and re-presentation of chaperoned peptides in antigen-presenting cells, providing a model of LRP1 targeting ligands into specialized endocytic compartments involved in re-secretion [24], [25]. On the other hand, the lysosomal targeting of LRP1 ligands such as the receptor associated protein (RAP) and activated α_2_-macroglobulin (α_2_M*) has been extensively used to study the role of LRP1 in the degradative pathway [26], [27]. Thus multiple ligands of LRP1 [28], [29] are targeted to different cellular compartments suggesting that specific binding affinities of the various ligands for LRP1 determine their intracellular destination.

In this study, we demonstrate that internalized TRL-derived apoE accumulates in LRP1 containing early endosomes, whereas RAP is targeted to (pre)-lysosomal compartments. Upon stimulation of HDL-induced recycling, apoE is no longer associated with LRP1-positive endosomes but targeted to LRP1-deficient, HDL-containing endosomes. LRP1 overexpression significantly stimulates HDL-induced apoE recycling, whereas down-regulation of LRP1 reduces the ability of HDL to promote apoE secretion. We conclude that LRP1 targets apoE into early endosomal recycling compartments to facilitate the intracellular formation and subsequent re-secretion of apoE enriched HDL particles.

## Results

### Intracellular localization of LRP1 and differential sorting of apoE and RAP

**Figure 1:**
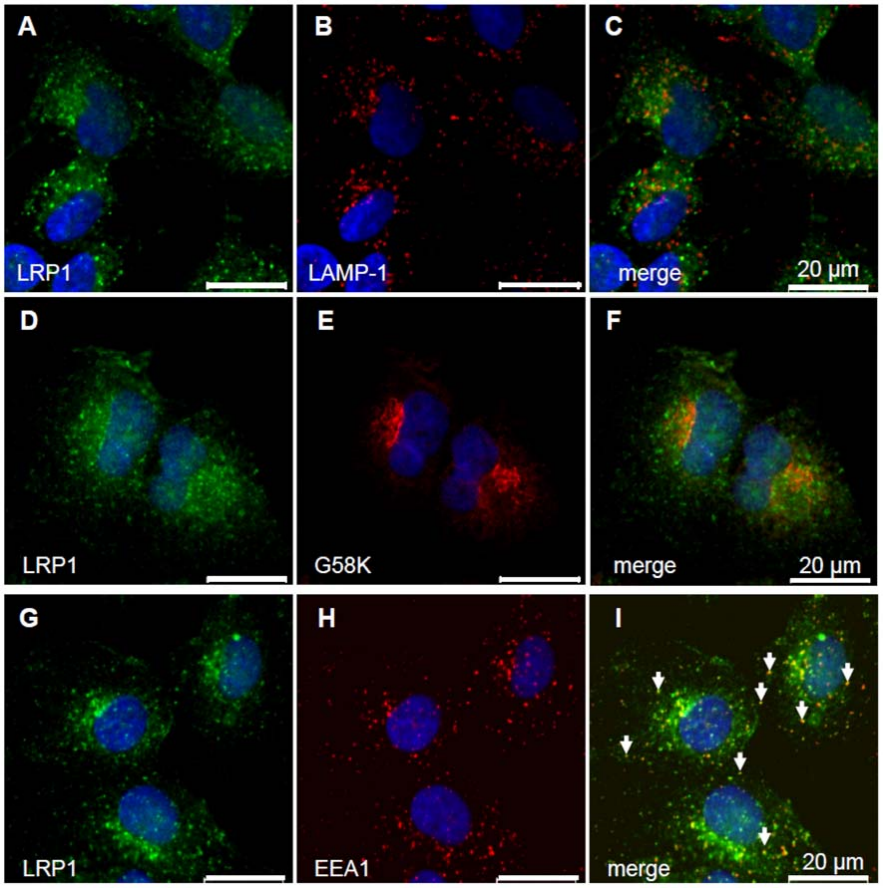
Endogenous LRP1 colocalizes with early endosomes markers. LRP1 was co-stained with LAMP-1 (A–C), the Golgi marker G58K (D–F) and EEA1 (G–I) in human hepatoma cells (HuH7) using respective antibodies. Confocal microscopy demonstrated a strong colocalization of LRP1 with the early endosome marker EEA1 in punctate peripheral and perinuclear endosomal structures (see arrows in the merged image, panel I). Nuclei appear in blue. Bar is 20 µm.

**Figure 2 pone-0029385-g002:**
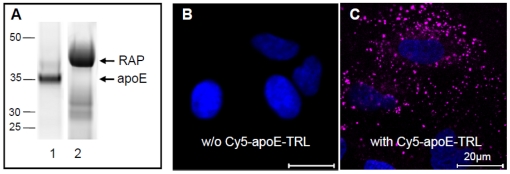
TRL-derived apoE is internalized into peripheral endosomal compartments. Cy5-apoE-TRL and Cy3-RAP were prepared and protein labelling was analyzed by SDS-PAGE and subsequent in-gel fluorescence detection (A). Incubation of HuH7 cells without (B) and with Cy5-apoE-TRL (C) for 30 min at 37°C resulted in a punctuated endosomal pattern indicating receptor-mediated endocytosis. Nuclei were visualized by DAPI and appear in blue. Bar is 20 µm.

**Figure 3 pone-0029385-g003:**
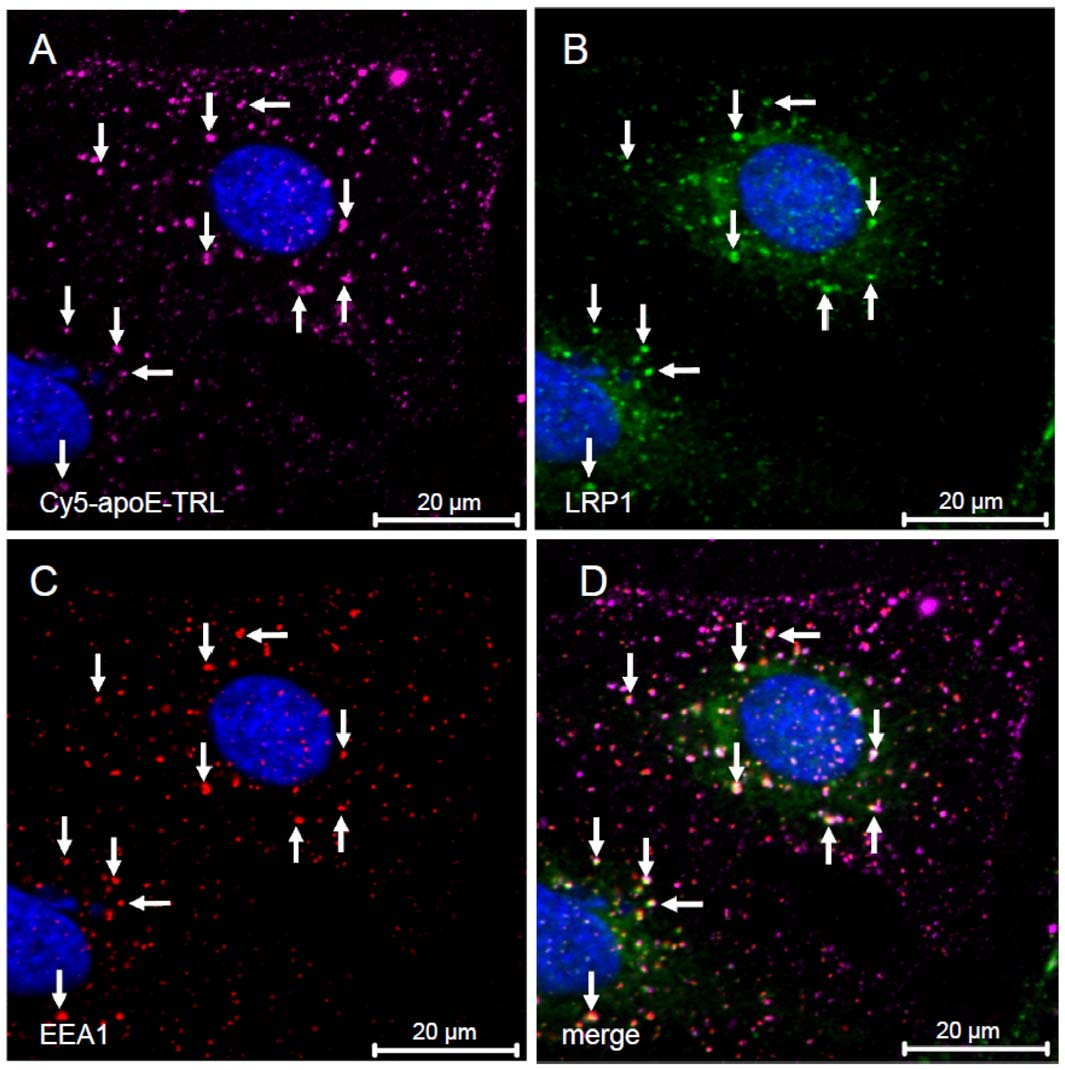
Internalized TRL-derived apoE co-localizes with LRP1 in EEA1-positive endosomes. HuH7 cells were incubated with Cy5-apoE-TRL for 10 min at 37°C. As indicated by the arrows, subsequent immunofluorescence analysis revealed co-localization of apoE (A) with LRP1 (B) in EEA1-positive endosomes (C). The merged image is shown in D. Nuclei were visualized by DAPI and appear in blue. Bar is 20 µm.

**Figure 4:**
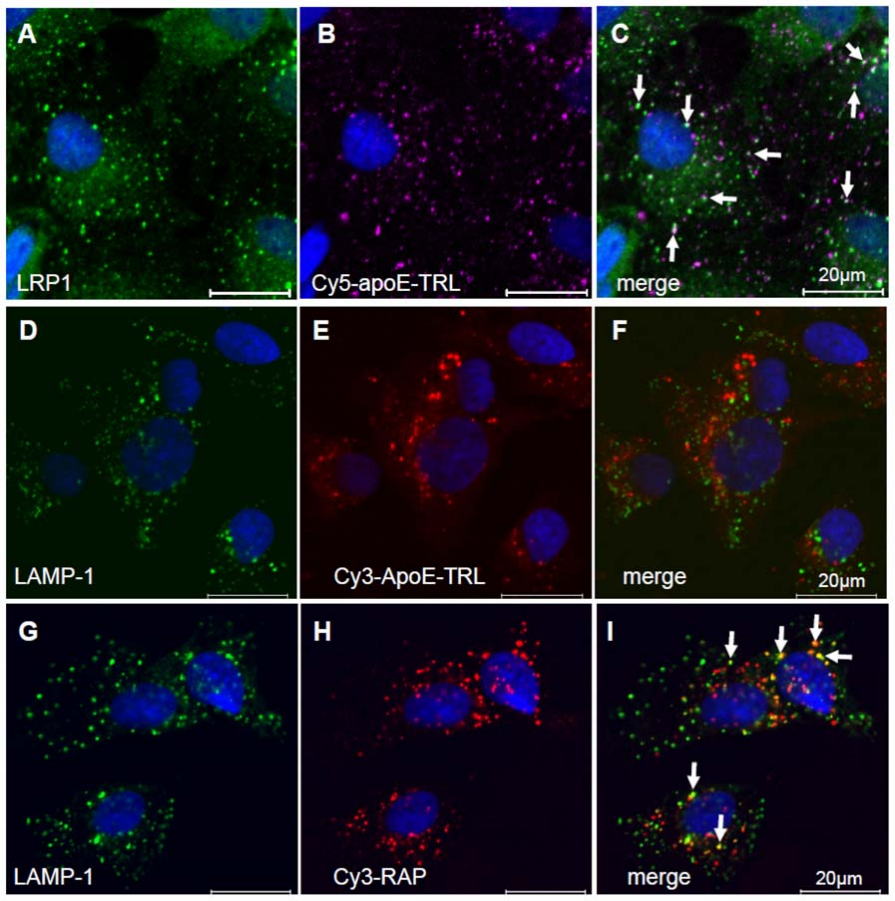
Internalized RAP but not TRL-derived apoE is sorted towards lysosomes. Incubation of HuH7 cells with Cy5-apoE-TRL or Cy3-RAP was performed for 30 min at 37°C. Subsequent confocal immunofluorescence analysis revealed colocalization of LRP1 with apoE (A–B, see arrows in merged image C). Internalized Cy3-apoE did not appear within lysosomes as indicated by a counterstain with LAMP-1 (D–F). In contrast, after 30 min Cy3-RAP co-localized with LAMP-1 (G–H, see arrows in the merged image I), indicating lysosomal targeting of the LRP1 ligand RAP. Nuclei were visualized by DAPI and appear in blue. Bar is 20 µm.

LRP1 has been shown to facilitate hepatic clearance of TRL. However, only a small proportion of LRP1 is present at the cell surface, whereas the majority of the receptor is found in intracellular, potentially endosomal compartments [7], [30]. To clarify the cellular localization of LRP1 in liver cells, we performed co-localization studies in human HuH7 hepatoma cells (Fig. 1). In agreement with previous findings, large amounts of LRP1 are localized predominantly in punctuate and partially perinuclear compartments (Fig. 1A, D and G). Importantly, LRP1 is not found in late endosomes or the Golgi-complex, as evidenced by the lack of colocalization with LAMP-1 (Fig. 1A–C) and the Golgi marker protein G58K (Fig. 1D–F), respectively, but it co-localizes with EEA1, a marker for early endosomes (Fig. 1G–I). Thus, in liver cells the major pool of LRP1 proteins is localized in the early endosomal compartment. To analyse if LRP1 is associated with internalized apoE in early endosomes we compared the intracellular localization of internalized and fluorescence-labelled apoE-TRL (Cy5-apoE-TRL) and RAP (Cy3-RAP) with LRP1. The efficiency and integrity of fluorescence-labelling of apoE-TRL and RAP was confirmed by SDS-PAGE and subsequent in-gel fluorescence detection (Fig. 2A). As shown previously, upon incubation of HuH7 hepatoma cells with Cy5-apoE-TRL, fluorescently labelled apoE is efficiently internalized (Fig. 2B–C) and appears within widely distributed peripheral endosomal compartments [17]. TRL-derived apoE is predominantly found in EEA1- and LRP1-positive endocytic vesicles 10 min (Fig. 3A–D) and 30 min (Fig. 4A–C) after incubation with Cy5-apoE-TRL indicating that substantial amounts of apoE are internalized via LRP1-mediated endocytosis and remains associated with LRP1. As shown previously [13], endocytosed apoE-TRL does not colocalize with LAMP-1 in (pre)-lysosomes (Fig. 4D–F). In contrast, RAP is efficiently delivered to lysosomal compartments under these conditions (Fig. 4G–I). These findings indicate that during TRL disintegration in early peripheral endosomes, apoE remains associated with LRP1 in EEA1-positive endosomes.

**Figure 5:**
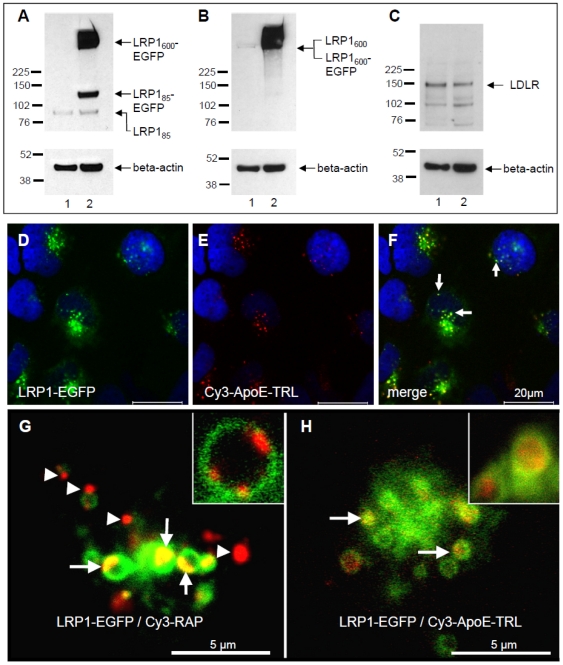
LRP1-dependent sorting of apoE and RAP. Cell lysates from EGFP (lane 1 in A, B and C) or LRP1-EGFP (lane 2 in A, B and C) transfected cells were subjected to SDS-PAGE, and Western blotting was performed with antibodies against the 85 kDa subunit of LRP1 (A), the 515 kDa subunit of LRP1 (B) and the LDLR (C). The endogenous LRP1 precursor protein is cleaved by furin into 515 kDa and 85 kDa fragments (shown in lane 1 of A and B). The recombinant LRP-EGFP can be detected at approximately 600 kDa (lane 2 in A and B) and for the cleaved 85 kDa fragment fused to EGFP at 115 kDa (LRP1_85_-EGFP; lane 2 in A). The overexpression of LRP1-EGFP expression slightly reduced the expression of LDLR (C). Western blotting using an antibody against beta actin verified equal protein loading. LRP1-EGFP transfected HEK293 cells were incubated with Cy3-apoE-TRL for 30 min. Confocal microscopy revealed strong colocalization of LRP1-EGFP and Cy3-apoE in endosomal compartments (D–E; see arrows in panel F; nuclei are stained with DAPI). High-magnification confocal live-cell microscopy of LRP1-EGFP expressing cells incubated with Cy3-RAP (G) or Cy3-apoE-TRL (H) revealed that RAP is still attached to the inner leaflet of the vesicular membrane and does not diffuse freely within the lumen (G, arrows and inlet). Additionally, RAP was detected in small, dense vesicles not containing LRP1 (G, arrowheads). In contrast, apoE was only present within LRP1-EGFP endosomes and evenly distributed inside the lumen (H, arrows). Bar is 5 (G–H) and 20 µm (D–F), respectively.

To further verify the role of LRP1 in apoE trafficking, we transfected human full-length LRP1 fused to EGFP (LRP1-EGFP) [31]. In order to achieve sufficient transfection rates with the 20 kb full-length LRP1 plasmid vector, HEK293 cells were used. Western blot analysis from transfected cells utilizing an antibody against the extracellular region of the 85 kDa LRP1 subunit distant to cytoplasmic fusion with EGFP, confirmed expression of ectopically expressed LRP1-EGFP fusion protein (Fig. 5A). Unprocessed endogenous and overexpressed LRP1 protein can be detected at approximately 515 and 600 kDa, respectively. Notably, the antibody revealed the presence of the small endogenous 85 kDa LRP1 and higher amounts of the 115 kD LRP1-EGFP subunit indicating furin cleavage [32] and appropriate processing of not only endogenous LRP1, but also the LRP1-EGFP fusion protein. Using an antibody against the 515 kDa subunit showed low levels of endogenous LRP1 protein, while considerable expression of the LRP1-EGFP fusion protein is detected (Fig. 5B). LRP1 deficiency correlates with elevated LDLR levels in liver of knockout mice [6], vice versa we observed minor reductions in LDLR protein levels upon ectopic LRP1 expression (Fig. 5C). Co-localization of LRP1-EGFP with cellular markers confirmed predominant localization of LRP1-EGFP in EEA1-positive endocytic compartments in HEK293 cells (data not shown). In support of the results described above (Fig. 4A–C) overexpression of LRP1-EGFP in HEK293 cells followed by a 30 min incubation with Cy3-apoE-TRL resulted in a strong colocalisation of LRP1 and internalized apoE in endocytic compartments (Fig. 5D–F).

To gain initial insights into LRP1-dependent sorting events in endosomes, we next compared the trafficking of Cy3-RAP and Cy3-apoE-TRL by confocal live-imaging (Fig. 5G–H). Video microscopy of LRP1-EGFP positive vesicles incubated with Cy3-RAP (Fig. 5G) or Cy3-apoE-TRL (Fig. 5H) for 30 min initially revealed the presence of small LRP1-containing vesicles (data not shown) followed by the fast appearance of Cy3-RAP in larger LRP1 endosomes, which were in average up to 1 µm in diameter. In agreement with the lysosomal trafficking of RAP, we observed a significant proportion of Cy3-RAP in vesicles not containing LRP1-EGFP. Furthermore, fluorescently-labelled RAP was absent from the lumen but associated with the inner leaflet of the endocytic, LRP1-EGFP positive membrane (see inlet in Fig. 5G), suggesting tight binding of RAP to LRP1 prior to their dissociation at later stages of the endocytic degradative pathway. Importantly, prominent differences were revealed when comparing apoE and RAP signals in LRP1-EGFP vesicles. While similar to Cy3-RAP, fluorescently labelled apoE-TRL rapidly appeared in large LRP1-containing endosomes; Cy3-apoE was found exclusively inside LRP1-EGFP positive vesicles in an evenly distributed fashion (Fig. 5H). These findings indicate LRP1-dependent differential ligand sorting and accumulation of apoE in peripheral early endosomes that prevent lysosomal targeting of endocytosed apoE.

### LRP1 influences the recycling rate of apoE

**Figure 6:**
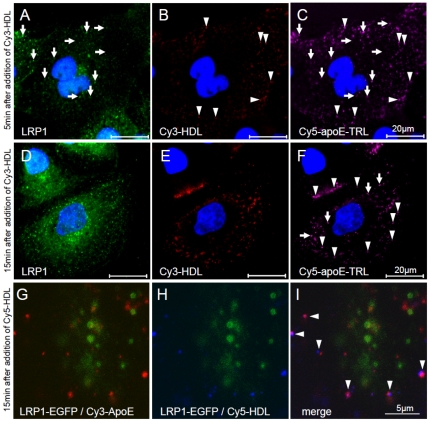
HDL stimulates the exit of apoE from LRP1- containing endosomes. HuH7 cells were incubated with Cy5-apoE-TRL for 20 minutes. ApoE recycling was then induced by Cy3-HDL. After 5 min (A–C) and 15 min (D–F), LRP1 (arrows) did no longer co-localize with Cy5-apoE which was instead found associated with Cy3-HDL (arrowheads; nuclei in blue). This was confirmed by high-magnification video-microscopy of LRP1-EGFP expressing HEK293 cells (G–I; due to short time-lapse between acquisition of red and blue channels, a minor signal offset due to endosomal movements was observed). Bar is 5 (G–I) and 20 µm (A–F), respectively.

Upon HDL-induced apoE recycling, apoE no longer colocalizes with LRP1 but is found together with HDL (Fig. 6A–F). Live-imaging confirmed that apoE leaves the LRP1-vesicles in the presence of HDL (Fig. 6G–I). Thus, HDL stimulates the exit of apoE from LRP1- containing endosomes which leads to the intracellular association of apoE with HDL.

**Figure 7:**
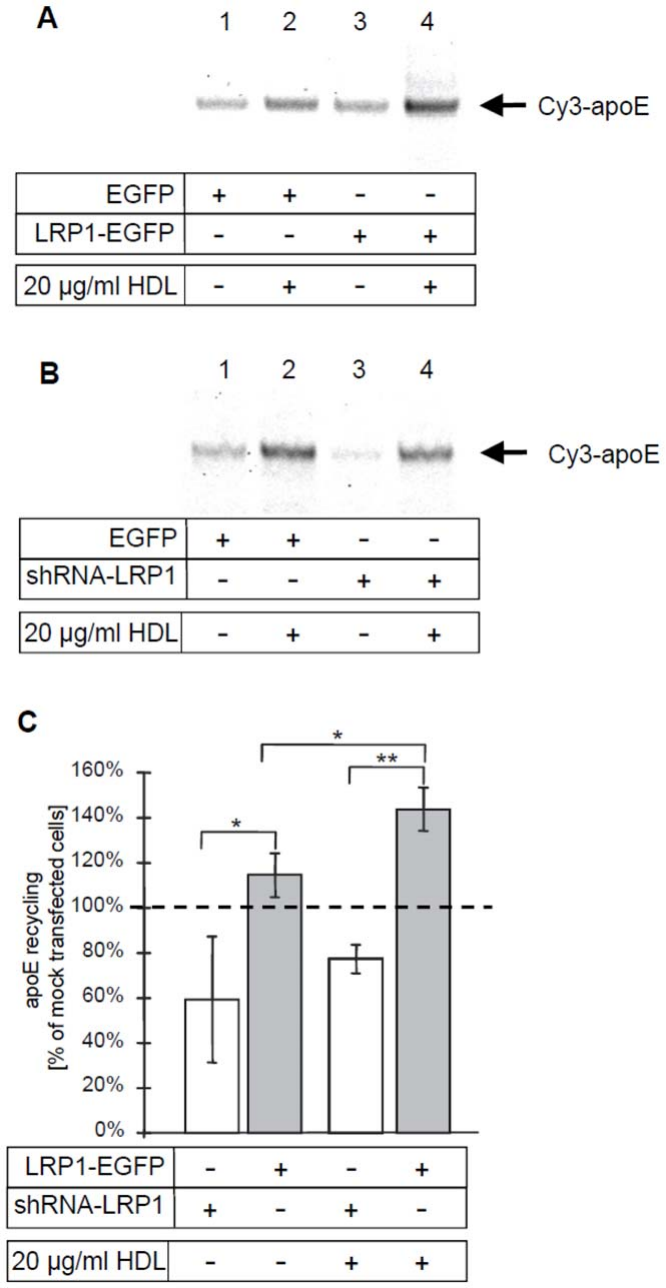
LRP1-dependent apoE recycling. HEK293 cells were transfected with pFB-LRP-EGFP, an shRNA vector against LRP1 or EGFP as control [31]. Pulse-chase experiments were performed by incubating HEK293 cells with Cy3-apoE-TRL for 60 min at 37°C. Cells were washed with heparin and incubated for additional 60 min at 37°C with media in the presence or absence of 20 µg/ml HDL_3_. Then cell culture media were harvested and the amount of re-secreted Cy3-apoE was determined by SDS-PAGE and subsequent in-gel fluorescence detection. The position of apoE is indicated. Representative gels with the corresponding apoE bands are shown for LRP1-EGFP overexpression (A) and reduced LRP1 expression (B). The highest and lowest intensity in each gel is represented as maximum black and white, respectively; therefore only intra-gel comparisons were performed. The quantification of these experiments is shown as percent recycling versus the corresponding mock transfected cells, which was set to 100% in order to ensure a valid comparison of the changes in apoE recycling between the different experiments (C). *: p<0.05, **: p<0.01 for Student's t-test (n≥4) ± S.E.M.

To quantify the involvement of LRP1 for HDL-induced apoE recycling, HEK293 cells expressing EGFP or LRP1-EGFP were incubated with Cy3-apoE-TRL for 60 min. Cell surface bound Cy3-apoE-TRL was removed by heparin, which was followed by an additional incubation ± HDL_3_ for 60 min. The amount of recycled Cy3-apoE was determined by SDS-PAGE of the chase media and subsequent in-gel fluorescence quantification (Fig. 7). Consistent with previous results [17], addition of HDL_3_ stimulated the recycling of Cy3-apoE two-fold compared to the control cells (Fig. 7A, lane 2 vs. 1). Importantly, the expression of LRP1-EGFP enhanced the HDL-induced recycling of Cy3-apoE (Fig. 7A, lane 4 vs. 2). In contrast, RNAi-mediated LRP1 knock-down [31] in HEK293 cells led to a decrease in HDL-induced apoE recycling (Fig. 7B). ApoE recycling in LRP1-EGFP and LRP1 knock-down cells was quantified in comparison to EGFP-transfected controls (Fig. 7C). The comparison of basal and HDL-dependent recycling revealed that LRP1-EGFP expression promoted HDL-induced apoE recycling (gray columns, approximately 40% increase) while LRP1 knock-down resulted in a 20% reduction in apoE recycling (white columns).

**Figure 8:**
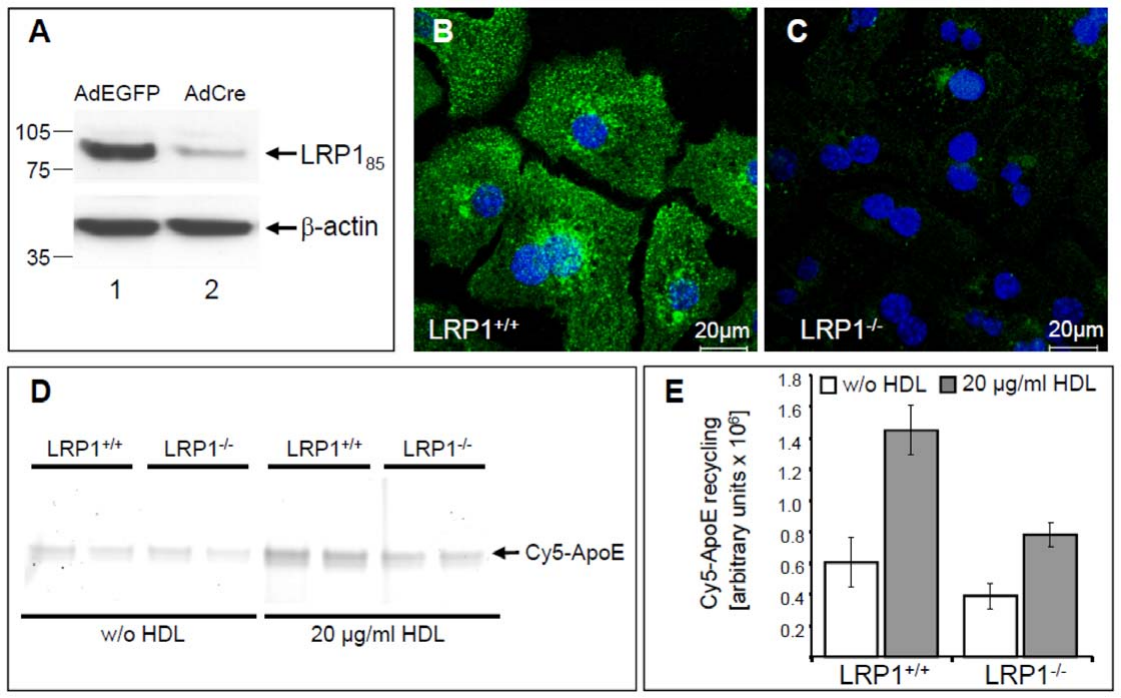
ApoE recycling is reduced in LRP1^−/−^ primary hepatocytes. LRP1*flox* mice were infected with AdEGFP or AdCre and three days after infection primary hepatocytes were isolated. Sixteen hours after seeding, the infection with AdCre resulted in a dramatic loss of LRP1 protein expression as determined by Western blotting (A) and indirect immunofluorescence (B–C). Pulse-chase experiments were performed by incubating LRP1-positive (LRP^+/+^) and LRP1–negative (LRP^−/−^) hepatocytes with Cy3-apoE-TRL for 60 min at 37°C. Cells were washed with heparin and incubated for additional 60 min at 37°C with media in the presence or absence (w/o) of 20 µg/ml HDL_3_. Then supernatants were harvested and the amount of re-secreted Cy3-apoE was determined by SDS-PAGE and subsequent quantification as described above. HDL-induced apoE recycling was reduced in LRP1^−/−^ cells as seen by in-gel fluorescence of chase media (D). Quantification of 4 independent experiments ± S.E.M. revealed a strong reduction of HDL-induced apoE recycling in LRP1^−/−^ hepatocytes ± HDL (E).

The quantitative role of LRP1 for apoE recycling was further evaluated in primary murine hepatocytes (Fig. 8). LRP1*flox* mice [6] were treated with an adenovirus encoding the Cre-recombinase (AdCre) in order to preclude hepatic LRP1 expression. Control animals were injected with a virus expressing EGFP (AdEGFP). Primary hepatocytes were prepared from both mice three days after infection, and Western blotting (Fig. 8A) and indirect immunofluorescence using an antibody against the 515 kDa LRP1 subunit (Fig. 8B–C) demonstrated the efficient knock-down of LRP1 expression in hepatocytes isolated from AdCre-treated mice. Importantly, the amount of basal, and even more pronounced, HDL-induced apoE-recycling was significantly reduced in LRP1^−/−^ cells (Fig. 8D–E). Taken together, while LRP1 knockdown lead to a decreased apoE recycling, the overexpression of LRP1 stimulated HDL-induced apoE recycling indicating that LRP1 expression correlated with apoE recycling efficiency.

## Discussion

The aim of the present study was to investigate the role of LRP1 for the intracellular targeting and recycling of apoE. Here we demonstrate that LRP1 strongly contributes to the retention and storage of apoE in early endosomes. Furthermore, LRP1 significantly reduces the delivery of apoE to the lysosomal compartment and prevents apoE degradation (Figs. 1–4). ApoE bound to LRP1 within early endosomes can be mobilized by HDL and is transported to a distinct population of early endosomes that contain HDL but not LRP1 (Fig. 5). As reported earlier, this process includes the temporary uptake of HDL and the association of recycled apoE with internalized HDL in early endosomes [17]. In contrast to apoE, which is trapped in LRP1-endosomes (Figs. 3, 4), RAP followed the lysosomal degradative pathway after lipoprotein receptor-mediated endocytosis (Fig. 4; [15], [27], [33]).

LRP2 (originally described as Megalin or gp330) is very similar to LRP1; it has a comparable molecular weight and also efficiently internalizes RAP [34]. LRP2 is expressed on the apical surfaces of absorptive epithelia including the yolk sac and the renal proximal tubules [35]. Czekay et al. presented a detailed analysis of the intracellular pathway and the sorting mechanisms of RAP after LRP2-mediated uptake [33]. The authors investigated the internalization of LRP2/RAP complexes and described the recycling of LRP2 to the cell surface, which in contrast to most other recycling receptors, including the LDL receptor family members, occurs from late endosomes. In these studies the pH-dependent release of the ligand appears to trigger the recycling of the receptor and since the LRP2/RAP association is rather pH-resistant, recycling occurs only at pH-values as low as in late endosomes. Here we show that similar to the LRP2/RAP complex, RAP remains bound to LRP1 at this point before its subsequent targeting to late endosomal compartments (Figs. 4, 5). In contrast, apoE under the same experimental conditions is released into the vesicular lumen (Fig. 5H) and is stored in LRP1-positive endosomes. Furthermore, both the RNAi-mediated down-regulation of LRP1 (Fig. 7) and the absence of LRP1 in primary murine hepatocytes (Fig. 8) resulted in decreased apoE recycling. Vice versa, LRP1 overexpression led to an enhanced HDL-induced apoE recycling (Fig. 7), indicating that LRP1 is most probably responsible for the differential intracellular targeting and recycling of internalized apoE. For the postprandial lipoprotein metabolism, this mechanism meets the physiological demands for an efficient uptake of TRL after a lipid-rich meal. Since TRL depend on apoE for an LRP1-mediated uptake into the liver, the HDL-bound apoE plasma pool is heavily used to transfer apoE to nascent TRL. The recycling of internalized apoE can be a valuable source for immediate replenishment [3]. The mechanistic fundament for a quick response of such a system is achieved by (a) the direct re-association of apoE with apoE-poor HDL during the recycling process and by (b) a short intracellular recycling route close to the plasma membrane, which only comprises early endosomes.

The physiological importance of intracellular LRP1-apoE storage and subsequent apoE recycling could also imply LRP1-dependent signal transduction pathways. Beyond endocytosis LRP1 regulates diverse cellular processes, for instance PDGF and Wnt-dependent signalling pathways, in an apoE-dependent manner [36]–[38]. These processes could be modified by apoE-mediated trapping of LRP1 and therefore apoE recycling might be a regulatory component of this signal transduction machinery. Hence this study might point to a novel concept in which apoE-mediated intracellular sequestration of lipoprotein receptors modulate their functions in regulating lipoprotein levels in the liver and signal transduction processes in peripheral tissues such as the central nervous system. In line with this hypothesis, it has recently been demonstrated that apoE isoforms differently modulate the function of apoE receptor 2 (apoER2), another LDL receptor family member, via apoER2 sequestration within intracellular compartments [39]. ApoE is a major susceptibility gene for the late onset of Alzheimer in which individuals with an apoE4 allele show an increased risk and an earlier onset of the disease. Chen et al. showed that impaired recycling of the apoE4 isoform in neuronal cells and in mice expressing the human apoE4 gene results in the trapping of postsynaptic ApoER2. As a consequence ApoER2 surface expression is reduced, which impairs NMDA receptor-mediated neurotransmission in apoE4 knock-in mice. Together with our study these findings point to a novel mechanism in which apoE-mediated intracellular sequestration of lipoprotein receptors modulate their respective functions.

**Figure 9:**
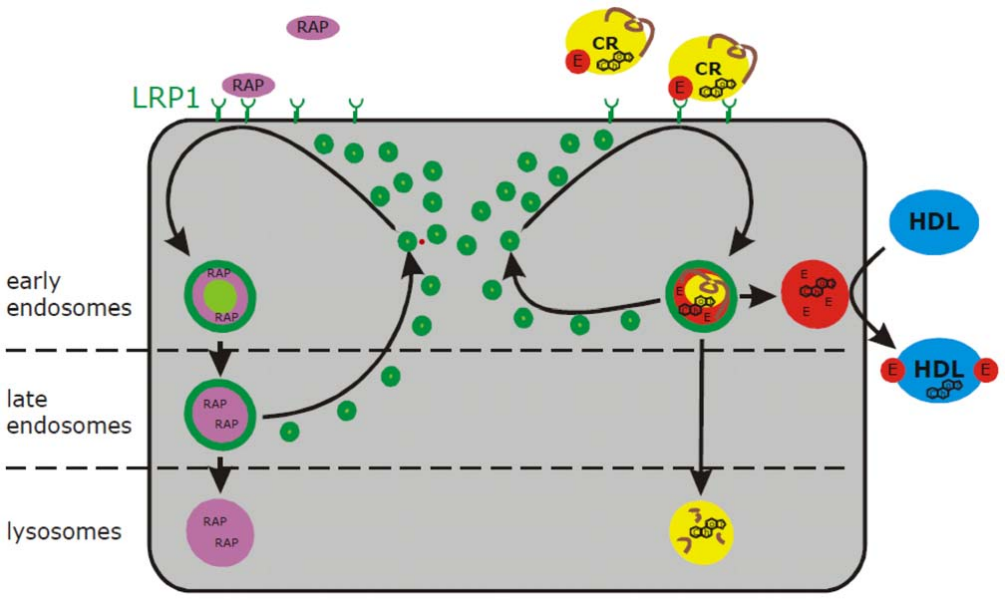
Model of LRP1-dependent uptake and apoE recycling. Left: RAP is internalized by LRP1, remains associated with the receptor in early endosomes and dissociates in late endosomes, from which LRP1 recycles back to the plasma membrane. RAP is degraded in the lysosome. Right: apoE-TRL are taken up by LRP1 via apoE and disintegrate in early endosomes. While apoE remains in early endosomes and LRP1 recycles back to the PM, the core particle is targeted to the lysosome. Upon stimulation with HDL, apoE separates from LRP1 and re-associates with HDL in early endosomes prior to re-secretion. See text for further details.

The results of our current work are summarized in Figure 9. Internalized RAP remains bound to LRP1 in early endosomes and is not released into the vesicular lumen. Once LRP1/RAP complexes arrive in late endosomes, they dissociate. LRP1 then returns to the plasma membrane while RAP is targeted to the lysosome. The uptake and trafficking of CR/apoE is strikingly different: LRP1 binds apoE, the particle disassembles after the uptake and the lipid core travels along early and late endosomes to the lysosome [13], [15]. ApoE is already released into the vesicular lumen and accumulates in LRP1-positive storage vesicles thereby escaping the degradation pathway. After the induction of recycling, apoE is immediately separated from LRP1 in early endosomes to re-associate with temporarily internalized HDL.

## Materials and Methods

### Antibodies and Reagents

Paraformaldehyde (PFA), glycine and BSA were purchased from Sigma. Mowiol was purchased from Calbiochem. DMEM, PBS, FCS, trypsin, penicillin and streptomycin were from Invitrogen (Gibco). Heparin (Liquemin) was purchased from Roche. Following antibodies were used: monoclonal antibody against EEA1 and LAMP-1 (BD Biosciences), monoclonal antibody against G58K and beta-actin (Sigma), anti-LRP1 (8G1 and 8B8; Progen) anti-LDLR (Progen), Cy2-conjugated donkey anti-rabbit F(ab′)2 fragments, Cy3-conjugated donkey anti-mouse F(ab′)2 fragments, Cy3-conjugated donkey anti-chicken F(ab′)2 fragments and horseradish peroxidase-conjugated goat anti-rabbit F(ab′)2 fragments were purchased from Jackson Immuno Research. Cy3 and Cy5 fluorescence protein labeling kit were from Amersham Biosciences.

### Cell culture and transfection

Human hepatoma cells (HuH7) and human embryo kidney (HEK-293) were grown in DMEM supplemented with 10% FCS and penicillin/streptomycin at 37°C in 5% CO_2_. To mediate LRP1 down-regulation and the transient expression of human recombinant LRP1 fused to the enhanced green fluorescent protein (LRP1-EGFP), HEK-293 were transfected in 6-wells using FuGENE 6 (Roche) as described [31]. To obtain LRP1-deficient and LRP1-expressing primary hepatocytes, LRP1flox mice (kindly provided by J. Herz, Dallas, TX) were tail-vein injected with 2.5·10^9^ IFU of an adenovirus expressing Cre recombinase (AdCre) or EGFP (AdEGFP), respectively. Primary mouse hepatocytes were prepared by liver perfusion, EDTA dissociation and centrifugation on a self-generating percoll gradient three days after infection as described [40]. Animal experiments were done in accordance with the guidelines of the Federation of American Societies for Experimental Biology (FASEB) and approved by the Department of Veterinary Affairs of the State of Hamburg.

### Ligand preparation

Recombinant RAP [41], apoE-deprived HDL_3_ (d = 1.125–1.21 g/ml) from normal healthy donors [17] and TRL from an apoCII-deficient patient were isolated as described [23]. Human recombinant apoE3 were expressed in BL21 Escherichia coli and purified by gel filtration chromatography. For immunofluorescence, 0.5 mg RAP was labelled with Cy3 to obtain Cy3-RAP. To obtain Cy3-apoE-TRL or Cy5-apoE-TRL, 100 µg of recombinant apoE3 were labelled with Cy3 or Cy5, respectively. Association of fluorescent labelled apoE with TRL was allowed for 60 min at 37°C and then Cy3-apoE-TRL or Cy5-apoE-TRL were re-isolated by ultracentrifugation. Fluorescent label was exclusively found in apoE as determined by SDS-PAGE followed by in-gel fluorescence analysis (see below). HDL_3_ apolipoproteins (1 mg) were labelled with Cy3 (Cy3-HDL) or Cy5 (Cy5-HDL) as described [17]. Fluorescent label was found predominantly in apoA-I (∼90–95%) as determined by SDS-PAGE, respectively (data not shown).

### Immunofluorescence and recycling experiments

For immunofluorescence experiments, human hepatoma cells were washed in PBS, fixed in 4% PFA and indirect immunfluorescence against LRP1 (polyclonal 377-4 provided by J. Herz, Dallas), LDLR, LAMP-1, G58K and EEA1 was performed. To analyse endocytosis, hepatoma cells were incubated with Cy3-apoE-TRL or Cy5-apoE-TRL (5 µg/ml) in DMEM+2% BSA at 37°C for 10 min or 30 min, respectively. Prior to fixation with 4% PFA, cells were washed in DMEM and cell surface-bound material was released by heparin treatment for 10 min at 4°C. To analyse intracellular targeting and recycling of apoE-TRL, hepatoma cells, transfected HEK-293 cells or primary hepatocytes were first incubated with Cy3-apoE-TRL or Cy5-apoE-TRL (5 µg/ml) in DMEM+2% BSA for 30 min at 37°C. Cells were washed in DMEM and treated with heparin for 10 min. To follow apoE recycling, cells were incubated for additional 0–60 min at 37°C in DMEM (0.1% BSA) ±20 µg/ml unlabeled or fluorescent-labelled HDL_3_ (Cy3-HDL_3_, Cy5-HDL_3_). Supernatants were harvested and processed as described below while cells were prepared for indirect immunofluorescence (see above). Confocal images were taken using a Zeiss LSM 510 (software version 3.0). For living cell microscopy, LRP1-EGFP expressing HEK-293 cells were incubated with Cy3-apoE-TRL, Cy5-apoE-TRL (5 µg/ml) or Cy3-RAP (10 µg/ml) in DMEM+2% BSA for 0–30 min at 37°C. To visualize HDL transport during apoE recycling, Cy3-apoE-TRL incubated cells were washed in DMEM and incubated for additional 0–30 min with 20 µg/ml Cy5-HDL_3_. Confocal images were taken every minute in the multitrack mode using optimized pinhole adjustment for each fluorochrome.

### SDS-PAGE, Western blot and protein quantification

To analyze the labelling of ligands, 10 µg of fluorescent RAP, HDL and apoE-TRL were separated by SDS-PAGE on 4–12% gradient gels (NuPAGE®, Invitrogen). Visualization was performed by in-gel scanning with the Typhoon 9410 (Amersham). To analyse lipoprotein receptor expression, cells were lysed in cell lysis buffer (2 mM CaCl_2_, 80 mM NaCl, 1% TritonX-100, 50 mM Tris/HCl, pH 8.0) and were analyzed using 4–12% gradient gels (Invitrogen). The expression of LRP1-EGFP in transfected HEK-293 cells was visualized by in-gel fluorescence detection. For western blotting, proteins were separated by SDS-PAGE in 4–12% gradient gels (Invitrogen), transferred to nitrocellulose and incubated with specific antibodies against different subunits of LRP1 (Progen, [15]), LDLR (Progen) and beta-actin (Sigma). The respective secondary horseradish peroxidase-labeled antibodies were from Jackson ImmunoResearch and signals were detected with enhanced chemiluminescence (ECL). For the quantification of apoE recycling, transfected HEK-293 cells or primary hepatocytes were incubated with fluorescent labelled apoE-TRL (1 µg/ml) for 60 min at 37°C, washed and incubated for additional 60 min at 37°C+/−20 µg/ml HDL_3_ in DMEM+0.1% BSA. The media were harvested, filtered (0.45 µm) and cleared by centrifugation at 14,000 g for 10 min. The supernatants were analyzed on 4–12% gradient gels and fluorescence-based in-gel quantification was performed by scanning with the Typhoon 9410 and analysis with the software ImageQuant 5.2 and/or FluorSep 2.2 (Molecular Dynamics).

## References

[1] Merkel M, Eckel RH, Goldberg IJ (2002). Lipoprotein lipase: genetics, lipid uptake, and regulation.. J Lipid Res.

[2] Bartelt A, Bruns OT, Reimer R, Hohenberg H, Ittrich H (2011). Brown adipose tissue activity controls triglyceride clearance.. Nat Med.

[3] Heeren J, Beisiegel U, Grewal T (2006). Apolipoprotein E recycling: implications for dyslipidemia and atherosclerosis.. Arterioscler Thromb Vasc Biol.

[4] Beisiegel U, Weber W, Ihrke G, Herz J, Stanley KK (1989). The LDL-receptor-related protein, LRP, is an apolipoprotein E-binding protein.. Nature.

[5] Beisiegel U, Weber W, Bengtsson-Olivecrona G (1991). Lipoprotein lipase enhances the binding of chylomicrons to low density lipoprotein receptor-related protein.. Proc Natl Acad Sci U S A.

[6] Rohlmann A, Gotthardt M, Hammer RE, Herz J (1998). Inducible inactivation of hepatic LRP gene by cre-mediated recombination confirms role of LRP in clearance of chylomicron remnants.. J Clin Invest.

[7] Laatsch A, Merkel M, Talmud PJ, Grewal T, Beisiegel U (2008). Insulin stimulates hepatic low density lipoprotein receptor-related protein 1 (LRP1) to increase postprandial lipoprotein clearance.. Atherosclerosis.

[8] Stanford KI, Bishop JR, Foley EM, Gonzales JC, Niesman IR (2009). Syndecan-1 is the primary heparan sulfate proteoglycan mediating hepatic clearance of triglyceride-rich lipoproteins in mice.. J Clin Invest.

[9] Williams KJ, Chen K (2010). Recent insights into factors affecting remnant lipoprotein uptake.. Curr Opin Lipidol.

[10] Havel RJ, Hamilton RL (2004). Hepatic catabolism of remnant lipoproteins: where the action is.. Arterioscler Thromb Vasc Biol.

[11] MacArthur JM, Bishop JR, Stanford KI, Wang L, Bensadoun A (2007). Liver heparan sulfate proteoglycans mediate clearance of triglyceride-rich lipoproteins independently of LDL receptor family members.. J Clin Invest.

[12] Out R, Kruijt JK, Rensen PC, Hildebrand RB, de Vos P (2004). Scavenger receptor BI plays a role in facilitating chylomicron metabolism.. J Biol Chem.

[13] Heeren J, Weber W, Beisiegel U (1999). Intracellular processing of endocytosed triglyceride-rich lipoproteins comprises both recycling and degradation.. J Cell Sci.

[14] Rensen PC, Jong MC, van Vark LC, van der BH, Hendriks WL (2000). Apolipoprotein E is resistant to intracellular degradation in vitro and in vivo. Evidence for retroendocytosis.. J Biol Chem.

[15] Heeren J, Grewal T, Jackle S, Beisiegel U (2001). Recycling of apolipoprotein E and lipoprotein lipase through endosomal compartments in vivo.. J Biol Chem.

[16] Swift LL, Farkas MH, Major AS, Valyi-Nagy K, Linton MF (2001). A recycling pathway for resecretion of internalized apolipoprotein E in liver cells.. J Biol Chem.

[17] Heeren J, Grewal T, Laatsch A, Rottke D, Rinninger F (2003). Recycling of apoprotein E is associated with cholesterol efflux and high density lipoprotein internalization.. J Biol Chem.

[18] Hasty AH, Plummer MR, Weisgraber KH, Linton MF, Fazio S (2005). The recycling of apolipoprotein E in macrophages: Influence of HDL and apolipoprotein AI.. J Lipid Res.

[19] Heeren J, Grewal T, Laatsch A, Becker N, Rinninger F (2004). Impaired recycling of apolipoprotein E4 is associated with intracellular cholesterol accumulation.. J Biol Chem.

[20] Rellin L, Heeren J, Beisiegel U (2008). Recycling of apolipoprotein E is not associated with cholesterol efflux in neuronal cells.. Biochim Biophys Acta.

[21] Davignon J, Gregg RE, Sing CF (1988). Apolipoprotein E polymorphism and atherosclerosis.. Arteriosclerosis.

[22] Roses AD (1996). Apolipoprotein E alleles as risk factors in Alzheimer's disease.. Annu Rev Med.

[23] Heeren J, Niemeier A, Merkel M, Beisiegel U (2002). Endothelial-derived lipoprotein lipase is bound to postprandial triglyceride-rich lipoproteins and mediates their hepatic clearance in vivo.. J Mol Med.

[24] Binder RJ, Han DK, Srivastava PK (2000). CD91: a receptor for heat shock protein gp96.. Nat Immunol.

[25] Binder RJ, Srivastava PK (2004). Essential role of CD91 in re-presentation of gp96-chaperoned peptides.. Proc Natl Acad Sci U S A.

[26] Herz J, Goldstein JL, Strickland DK, Ho YK, Brown MS (1991). 39-kDa protein modulates binding of ligands to low density lipoprotein receptor-related protein/alpha 2-macroglobulin receptor.. J Biol Chem.

[27] Willnow TE, Orth K, Herz J (1994). Molecular dissection of ligand binding sites on the low density lipoprotein receptor-related protein.. J Biol Chem.

[28] Herz J, Strickland DK (2001). LRP: a multifunctional scavenger and signaling receptor.. J Clin Invest.

[29] Lillis AP, Mikhailenko I, Strickland DK (2005). Beyond endocytosis: LRP function in cell migration, proliferation and vascular permeability.. J Thromb Haemost.

[30] Harasaki K, Lubben NB, Harbour M, Taylor MJ, Robinson MS (2005). Sorting of major cargo glycoproteins into clathrin-coated vesicles.. Traffic.

[31] Laatsch A, Ragozin S, Grewal T, Beisiegel U, Heeren J (2004). Differential RNA interference: replacement of endogenous with recombinant low density lipoprotein receptor-related protein (LRP).. Eur J Cell Biol.

[32] Willnow TE, Moehring JM, Inocencio NM, Moehring TJ, Herz J (1996). The low-density-lipoprotein receptor-related protein (LRP) is processed by furin in vivo and in vitro.. Biochem J.

[33] Czekay RP, Orlando RA, Woodward L, Lundstrom M, Farquhar MG (1997). Endocytic trafficking of megalin/RAP complexes: dissociation of the complexes in late endosomes.. Mol Biol Cell.

[34] Willnow TE, Goldstein JL, Orth K, Brown MS, Herz J (1992). Low density lipoprotein receptor-related protein and gp330 bind similar ligands, including plasminogen activator-inhibitor complexes and lactoferrin, an inhibitor of chylomicron remnant clearance.. J Biol Chem.

[35] Muller D, Nykjaer A, Willnow TE (2003). From holoprosencephaly to osteopathology: role of multifunctional endocytic receptors in absorptive epithelia.. Ann Med.

[36] Terrand J, Bruban V, Zhou L, Gong W, El Asmar Z (2009). LRP1 controls intracellular cholesterol storage and fatty acid synthesis through modulation of Wnt signaling.. J Biol Chem.

[37] Boucher P, Li WP, Matz RL, Takayama Y, Auwerx J (2007). LRP1 functions as an atheroprotective integrator of TGFbeta and PDFG signals in the vascular wall: implications for Marfan syndrome.. PLoS ONE.

[38] Boucher P, Gotthardt M, Li WP, Anderson RG, Herz J (2003). LRP: role in vascular wall integrity and protection from atherosclerosis.. Science.

[39] Chen Y, Durakoglugil MS, Xian X, Herz J (2010). ApoE4 reduces glutamate receptor function and synaptic plasticity by selectively impairing ApoE receptor recycling.. Proc Natl Acad Sci U S A.

[40] Meredith MJ (1988). Rat hepatocytes prepared without collagenase: prolonged retention of differentiated characteristics in culture.. Cell Biol Toxicol.

[41] Kounnas MZ, Argraves WS, Strickland DK (1992). The 39-kDa receptor-associated protein interacts with two members of the low density lipoprotein receptor family, alpha 2-macroglobulin receptor and glycoprotein 330.. J Biol Chem.

